# Effects of Kiperin Elixea, a multicomponent antioxidant supplement, on redox homeostasis and longevity-associated gene expression in human epithelial cell models

**DOI:** 10.3389/fragi.2026.1843098

**Published:** 2026-06-15

**Authors:** Lutfiye Karcıoğlu Batur, Ahsen Pektas, Buse Aslan, Nermin Akcali

**Affiliations:** 1 Department of Molecular Biology and Genetics, Faculty of Engineering and Natural Sciences, Biruni University, Istanbul, Türkiye; 2 Biruni University Research Center (B@MER), Biruni University, Istanbul, Türkiye

**Keywords:** Kiperin Elixea, longevity, nicotinamide mononucleotide (NMN), nutraceuticals, redox homeostasis

## Abstract

**Introduction:**

Aging is characterized by declining NAD^+^ levels, mitochondrial dysfunction and disruption of redox homeostasis. Multi-component formulations targeting interconnected hallmarks of aging have emerged as potential geroprotective strategies.

**Methods:**

We evaluated the molecular and functional effects of Elixea, an NMN (nicotinamide mononucleotide)-based formulation containing NAD^+^ precursors (NMN, NADH), mitochondrial cofactors (CoQ10, PQQ), and antioxidants (resveratrol, glutathione), in human epithelial cell models. hTERT-HME1 and HaCaT cells were treated with 0.1 μg/mL Elixea for 24 h. Cellular viability, regenerative kinetics, total antioxidant status (TAS), total oxidant status (TOS), and expression of longevity-associated genes were assessed.

**Results:**

Elixea maintained normal cell viability and migration dynamics, supporting the preservation of basal epithelial function. In hTERT-HME1 cells, treatment significantly improved redox homeostasis, as evidenced by increased TAS and reduced TOS levels. Gene expression analysis revealed significant upregulation of *IL10, FOXO3A, APOE, GPX1*, and *FOXO1A*, indicating activation of pathways associated with antioxidant defense, anti-inflammatory signaling, and metabolic regulation. In HaCaT cells, responses were more selective, including upregulation of *APOE, FOXO3A*, and *RUVBL1*.

**Discussion:**

Collectively, these findings suggest that Elixea modulates conserved longevity-associated pathways in a cell type-dependent manner, promoting cellular resilience without evidence of cytotoxicity or overt stress-associated responses. This study provides mechanistic evidence supporting the geroprotective potential of multi-component interventions targeting redox and metabolic networks in epithelial systems.

## Introduction

Nicotinamide mononucleotide (NMN) has recently emerged as a widely discussed dietary supplement ingredient due to its role in NAD^+^ metabolism, a central hub linking cellular energy production, redox homeostasis, and stress-response signaling (Kulkarni and Brookes, 2019; Verdin, 2015). Declining NAD^+^ availability is a well-recognized hallmark of aging, contributing to impaired mitochondrial function, reduced metabolic flexibility, and increased susceptibility to cellular stress (Chini et al., 2024; López-Otín et al., 2023). Accordingly, NMN-containing formulations are increasingly positioned as longevity-associated interventions aimed at restoring metabolic fitness and delaying age-associated functional decline (Tarbiat et al., 2024).

In addition to NAD^+^ precursors, several supplement formulations incorporate antioxidants and mitochondrial cofactors to target interconnected hallmarks of aging, including oxidative stress, mitochondrial dysfunction, and chronic low-grade inflammation (Ferrucci and Fabbri, 2018; Franceschi and Campisi, 2014; Liguori et al., 2018; Xu et al., 2025). Mitochondrial integrity is particularly critical in epithelial tissues, where age-related decline contributes to functional deterioration and impaired tissue maintenance (Haas, 2019; Kloepper et al., 2015; Rath et al., 2018; Wikramanayake et al., 2022). Within this context, compounds such as coenzyme Q10 (CoQ10) and pyrroloquinoline quinone (PQQ) support mitochondrial electron transport and biogenesis (Hidalgo-Gutiérrez et al., 2021; Hwang et al., 2020; Kuo et al., 2015), while resveratrol and glutathione contribute to redox regulation through both direct antioxidant activity and activation of endogenous defense pathways (Aquilano et al., 2014; Barcelos and Haas, 2019).

Elixea is a multi-component supplement combining NMN, NADH, resveratrol, glutathione, CoQ10, and PQQ, thereby targeting two major axes of aging biology: (i) mitochondrial bioenergetic efficiency and redox control, and (ii) antioxidant buffering capacity and stress-adaptive signaling. Despite the growing popularity of such formulations, mechanistic evidence remains limited regarding whether these combinations induce coordinated cellular responses consistent with enhanced resilience and longevity-associated pathways. Importantly, this combinatorial formulation is consistent with the geroscience hypothesis, which posits that simultaneous targeting of multiple interconnected hallmarks of aging yields more effective biological outcomes than single-pathway interventions (Panchin A, et al., 2024). Given the tight mechanistic coupling between mitochondrial dysfunction, redox imbalance, and chronic low-grade inflammation, the integration of NAD^+^ precursors, mitochondrial cofactors, and antioxidant molecules within a single formulation may enable coordinated modulation of aging-related networks rather than isolated molecular effects. Such multi-target approaches are increasingly recognized as a prerequisite for achieving robust and sustained improvements in cellular resilience.

In the context of healthy aging, cellular resilience is defined by the coordinated regulation of oxidative stress resistance, metabolic adaptation, inflammatory tone, and maintenance of genomic stability (López-Otín et al., 2023; Rando et al., 2025). *FOXO (forkhead box O)* transcription factors, particularly *FOXO1A (forkhead box O1A)* and *FOXO3A (forkhead box O3A)* are central regulators of these processes, integrating redox signaling, autophagy, and metabolic homeostasis (Martins et al., 2016; Rodriguez-Colman et al., 2024; Zhao and Liu, 2021). These pathways are closely linked to NAD^+^-dependent signaling networks, including sirtuin-mediated regulation (Verdin, 2015). In parallel, antioxidant enzymes such as *GPX1 (glutathione peroxidase 1)* play a critical role in maintaining intracellular redox homeostasis by detoxifying reactive oxygen species (Huang et al., 2025; Mandraffino et al., 2012).

Chronic low-grade inflammation (inflammaging) represents another key hallmark of aging, contributing to tissue dysfunction and age-related diseases (Ferrucci and Fabbri, 2018; Franceschi and Campisi, 2014). In this context, *IL10 (interleukin 10),* is of particular interest as an anti-inflammatory cytokine capable of modulating inflammatory signaling and promoting tissue homeostasis (Dagdeviren et al., 2017; Kinzenbaw et al., 2013). Additionally, *APOE (apolipoprotein E),* has been implicated in metabolic regulation and lipid homeostasis, linking cellular metabolism to aging-related processes. Conversely, *TP53* serves as a key regulator of genomic integrity and stress responses, allowing evaluation of whether an intervention induces damage-associated pathways (Pandey et al., 2025), while *RUVBL1 (RuvB-like AAA ATPase 1)* participates in broader cellular regulatory networks associated with homeostasis.

Within this framework, the present study aimed to evaluate whether Elixea induces cellular responses consistent with longevity-associated mechanisms in epithelial cells. Using hTERT- HME1 and HaCaT models, we assessed functional outputs such as cell viability and migration, together with global redox homeostasis (TAS/TOS) and gene expression profiles related to oxidative stress, inflammation, and metabolic regulation. This integrated approach enables a systems-level evaluation of how a multi-component NMN-based formulation may influence redox homeostasis and stress-response pathways in epithelial cell systems.

## Results

### Effects of Elixea on cell viability

**FIGURE 1:**
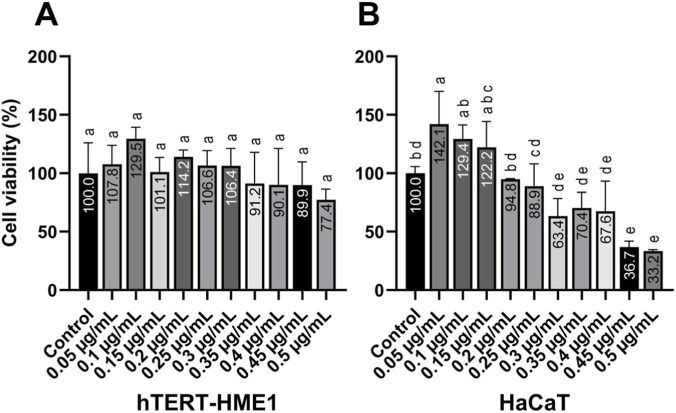
Effects of the Elixea on cell viability in hTERT-HME1 and HaCaT cells for 24 h (n = 3). **(A)** Cell viability of hTERT-HME1 cells following treatment with increasing concentrations (0.05–0.5 μg/mL) of the compound. **(B)** Cell viability of HaCaT cells under the same treatment conditions. Cell viability was expressed as a percentage relative to the control group, which was set to 100%. Data are presented as mean ± SD. Statistical analysis was performed using one-way ANOVA followed by Tukey’s multiple comparison test. Different letters above the bars indicate statistically significant differences between groups, whereas groups sharing the same letter are not significantly different (p < 0.05).

The effects of Elixea on cell viability were evaluated in hTERT-HME1 and HaCaT cells (Figure 1) for 24 h. As shown in Figure 1A, treatment of hTERT-HME1 cells with increasing concentrations of Elixea (0.05–0.5 μg/mL) did not result in any statistically significant changes compared to the control group (p > 0.05). Cell viability remained stable across all concentrations, indicating that Elixea does not exert any detrimental effects on normal epithelial cell viability within the tested range.

In contrast, Figure 1B demonstrates that HaCaT cells responded to Elixea in a concentration-dependent manner. Notably, low concentrations (0.05–0.15 μg/mL) led to an increase in cell viability compared to the control group, suggesting a stimulatory effect on cell proliferation. This proliferative response was evident at 0.1 μg/mL and reached its maximum at 0.05 μg/mL. At intermediate concentrations (0.2–0.35 μg/mL), cell viability gradually declined toward control levels, while higher concentrations (≥0.4 μg/mL) resulted in reduced viability. Based on these findings, 0.1 μg/mL was selected as the optimal concentration for subsequent experiments, as it supported enhanced cell viability in HaCaT cells while maintaining a non-cytotoxic profile in hTERT-HME1 cells.

Taken together, Elixea appears to increase cell viability at lower concentrations while remaining non-toxic to normal epithelial cells and was therefore used at low doses in subsequent experiments.

### Effects of Elixea on regenerative kinetics

**FIGURE 2:**
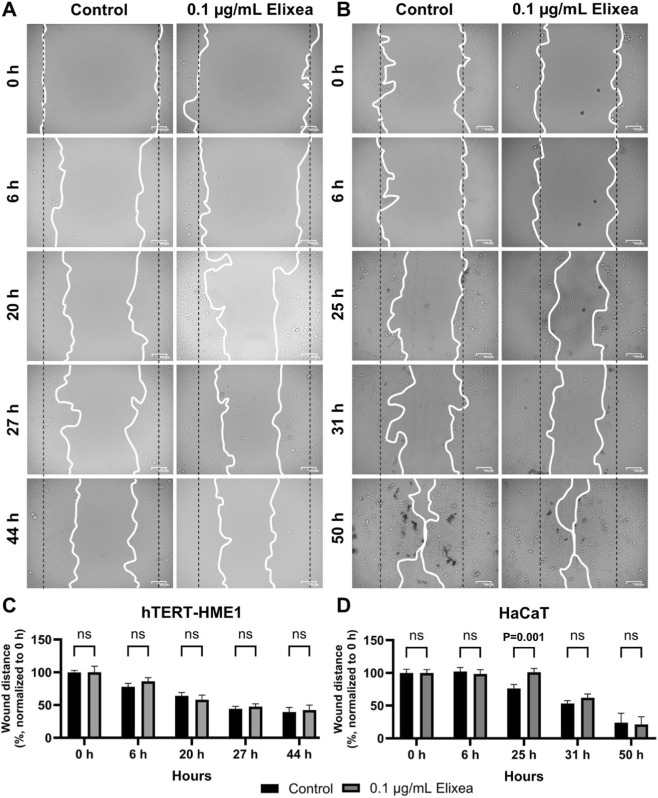
Effects of Elixea on cell migration assessed by wound healing assay in hTERT-HME1 and HaCaT cells (n = 3). **(A)** Representative inverted microscope images of wound closure in hTERT-HME1 cells at indicated time points (0, 6, 20, 27, and 44 h) for control and 0.1 μg/mL Elixea-treated groups. **(B)** Representative wound healing images of HaCaT cells at indicated time points (0, 6, 25, 31, and 50 h). **(C)** Quantitative analysis of wound closure in hTERT-HME1 cells. **(D)** Quantitative analysis of wound closure in HaCaT cells. For all analyses, the wound width at 0 h was normalized to 100% and subsequent time points were expressed as percentages relative to baseline. Data are presented as mean ± SD. Statistical significance was evaluated using Student’s t-test (ns: not significant). Scale bar: 100 µm.

The effects of Elixea on cell migration were shown in Figure 2. Consistent with the cell viability findings, which showed no adverse effects of Elixea on hTERT-HME1 cells, Figure 2A and C demonstrates that hTERT-HME1 cells exhibited a gradual reduction in wound width over time in both control and treated groups, reflecting normal migratory behavior. No statistically significant differences were observed between the groups at any time point (p > 0.05), indicating that Elixea at 0.1 μg/mL does not affect the migration capacity of these cells.

In HaCaT cells, where low-dose Elixea treatment enhanced cell viability, a distinct pattern was observed in migration (Figures 2B,D). While no significant differences were detected at early time points (6 h), a significant delay in regenerative kinetics was observed at 25 h in the Elixea-treated group compared to the control (p < 0.01). However, this effect was transient, as no statistically significant differences were found at later time points (31 and 50 h).

### Effects of Elixea on redox homeostasis

**FIGURE 3:**
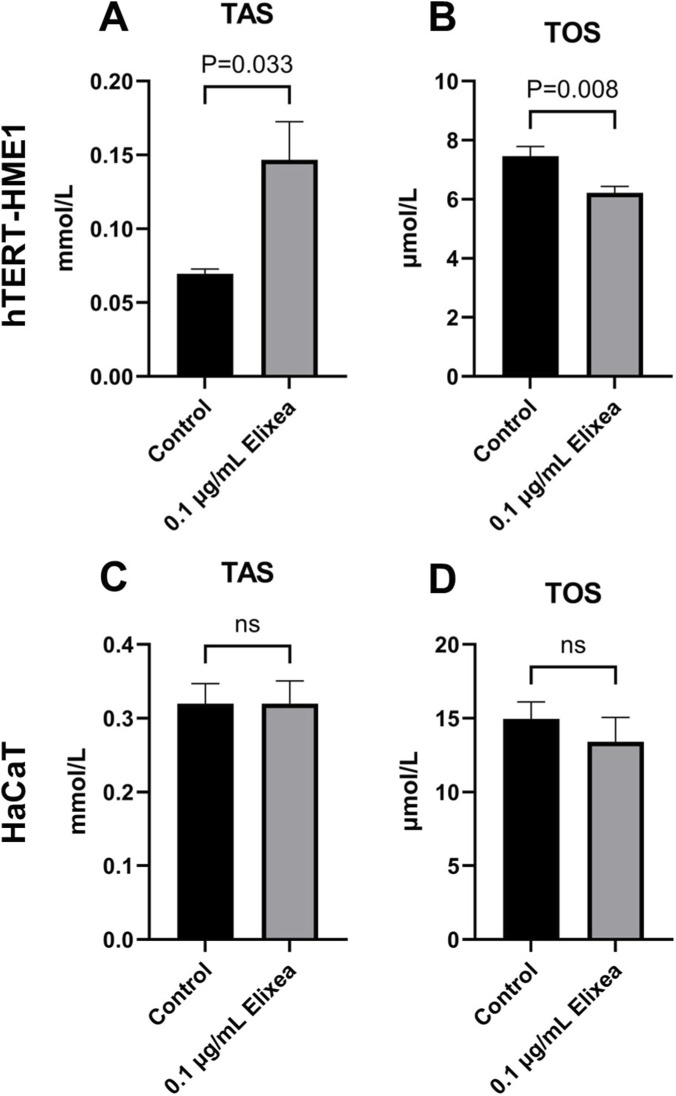
Effects of Elixea on oxidative status in hTERT-HME1 and HaCaT cells for 24 h (n = 3). **(A)** TAS levels in hTERT-HME1 cells. **(B)** TAS levels in HaCaT cells. **(C)** TOS levels in hTERT- HME1 cells. **(D)** TOS levels in HaCaT cells. Cells were treated with 0.1 μg/mL Elixea and compared to untreated control groups. Data are presented as mean ± SD. Statistical analysis was performed using Student’s t-test (ns: not significant).

The effects of Elixea on oxidative status were evaluated by measuring TAS and TOS in hTERT- HME1 and HaCaT cells (Figure 3) for 24 h. In hTERT-HME1 cells, Elixea treatment significantly reduced TOS levels compared to the control group (p = 0.008), indicating a decrease in oxidative burden. In parallel, TAS levels were significantly increased following treatment (p = 0.033) (Figures 3A,B). Together, these findings suggest that Elixea enhances the antioxidant capacity while reducing oxidative stress in hTERT-HME1 cells. In HaCaT cells, no statistically significant differences were observed between control and Elixea-treated groups for either TOS or TAS levels (p > 0.05) (Figures 3C,D). Although minor variations were detected, overall oxidative and antioxidant status remained comparable between groups.

Consistent with the cell viability results showing that Elixea does not exert cytotoxic effects in hTERT-HME1 cells and supports viability in HaCaT cells at low concentrations, these findings indicate that Elixea contributes to maintaining redox homeostasis, particularly by enhancing antioxidant defenses in hTERT-HME1 cells.

### Effects of Elixea on longevity-associated gene expression

**FIGURE 4:**
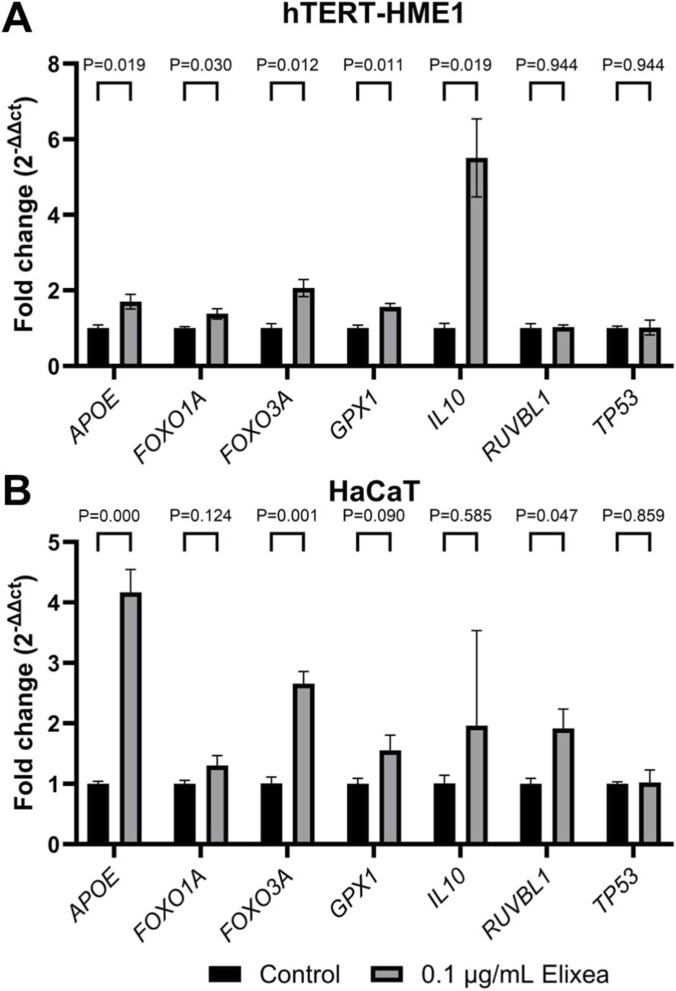
Effects of Elixea on gene expression levels in hTERT-HME1 and HaCaT cells (n = 3). **(A)** Relative mRNA expression of *APOE*, *FOXO1A*, *FOXO3A*, *GPX1*, *IL10*, *RUVBL1* and *TP53* in hTERT-HME1 cells following treatment with 0.1 μg/mL Elixea (24 h). **(B)** Relative mRNA expression of the same genes in HaCaT cells under identical conditions. Expression levels were calculated using the 2^−ΔΔCt^ method, with *GAPDH* as the reference gene and normalized to the control group, which was set to 1. Data represent independent replicates (n = 3) and are presented as mean ± SD. Statistical analysis was performed using Student’s t-test; p-values are indicated above the bars.

The effects of Elixea on the expression of genes associated with oxidative stress, inflammation, and cellular regulation were evaluated in hTERT-HME1 and HaCaT cells (Figure 4). In hTERT- HME1 cells, Elixea treatment resulted in significant upregulation of several genes. Expression levels of *APOE* (1.70-fold, p = 0.019), *FOXO1A* (1.38-fold, p = 0.030), and *FOXO3A* (2.07-fold, p = 0.012) were significantly increased compared to the control group. Similarly, the antioxidant enzyme *GPX1* showed a significant upregulation (1.56-fold, p = 0.011), consistent with the observed increase in TAS and decrease in TOS levels. In addition, *IL10* expression was markedly elevated (5.51-fold, p = 0.019), suggesting an anti-inflammatory response. In contrast, no significant changes were observed in *RUVBL1* or *TP53* expression (p > 0.05).

In HaCaT cells, Elixea treatment also induced notable changes in gene expression. A strong upregulation of *APOE* (4.17-fold, p < 0.001) and *FOXO3A* (2.66-fold, p = 0.001) was observed. *RUVBL1* expression was also significantly increased (1.91-fold, p = 0.047). Although *FOXO1A* and *GPX1* showed an increasing trend, these changes were not statistically significant. No significant difference was detected in *IL10* and *TP53* expressions.

Consistent with the TAS/TOS findings, the upregulation of antioxidant-related genes such as *FOXO3A* and *GPX1* in hTERT-HME1 cells supports the enhancement of cellular antioxidant capacity. In HaCaT cells, the prominent increase in *APOE* and *FOXO3A* expression aligns with the observed increase in cell viability at low concentrations, suggesting that Elixea may modulate pathways associated with cellular metabolism and stress response.

## Discussion

In this study, we evaluated the biological effects of Elixea, a multi-component supplement containing resveratrol, glutathione, coenzyme Q10 (CoQ10), nicotinamide mononucleotide (NMN), NADH and pyrroloquinoline quinone (PQQ) in hTERT-HME1 epithelial cells and HaCaT keratinocyte cells. Collectively, our findings indicate that Elixea primarily modulates redox homeostasis and longevity-associated transcriptional pathways rather than directly altering migratory behavior or inducing stress-associated responses.

Although no statistically significant change in cell viability was observed in hTERT-HME1 cells, the stable viability profile across all tested concentrations, together with a slight upward tendency at lower doses, may reflect preserved or modestly enhanced cellular metabolic competence rather than cytotoxic stress. In contrast, HaCaT cells exhibited a more pronounced increase in viability at lower concentrations, suggesting a cell type-dependent response to Elixea. Mitochondrial dysfunction and NAD^+^ decline are recognized hallmarks of aging (Chini et al., 2024; López-Otín et al., 2023; Mohamad Ishak et al., 2024; Rando et al., 2025). NMN and NADH are directly involved in NAD^+^ metabolism (Kulkarni and Brookes, 2019), which regulates sirtuin activity and cellular stress responses (Verdin, 2015). In parallel, CoQ10 and PQQ contribute to mitochondrial electron transport efficiency and mitochondrial biogenesis (Hidalgo- Gutiérrez et al., 2021; Hwang et al., 2020; Kuo et al., 2015; Mecit et al., 2023). Maintenance of mitochondrial integrity is central to cellular longevity, particularly in epithelial tissues such as the skin (Kloepper et al., 2015; Rath et al., 2018), where mitochondrial decline contributes to age-associated functional deterioration (Haas, 2019; Wang et al., 2025; Wikramanayake et al., 2022). Within this context, the absence of cytotoxicity in hTERT-HME1 cells alongside enhanced viability in HaCaT cells may indicate that Elixea supports cellular bioenergetic resilience and viability in a context-dependent manner, rather than acting as a direct proliferative stimulus across all epithelial cell types.

Importantly, Elixea did not significantly alter wound closure kinetics in hTERT-HME1 cells, indicating that basal migratory capacity was preserved. In HaCaT cells, a transient delay in regenerative kinetics was observed at an intermediate time point. However, this effect was not sustained and overall migration dynamics remained comparable between groups. The wound healing assay was included as a functional readout to determine whether Elixea alters basal epithelial migratory behavior in addition to its effects on viability, redox status, and gene expression. The transient delay observed in HaCaT cells should therefore be interpreted cautiously and does not support a definitive conclusion regarding enhanced or more controlled repair; rather, the overall findings indicate that Elixea did not cause a sustained impairment of epithelial migration under the tested conditions. Rather than promoting acute repair signaling, these findings suggest maintenance of basal epithelial homeostasis. In longevity biology, excessive proliferation or stress-induced regeneration is not necessarily beneficial (Campisi, 2013; López-Otín et al., 2013). Instead, the maintenance of redox homeostasis and metabolic stability is considered more relevant for long-term tissue integrity (Arnal-Forné and Borrás, 2025; Tullet et al., 2008). The largely unchanged migratory profile, together with enhanced antioxidant status and FOXO-associated signaling, supports a homeostatic and resilience-oriented mode of action.

A key finding of this study was the modulation of oxidative status in a cell type-dependent manner. In hTERT-HME1 cells, Elixea treatment resulted in a significant increase in total antioxidant status accompanied by a reduction in total oxidant status, indicating an overall improvement in redox homeostasis. In contrast, no significant changes were observed in HaCaT cells, suggesting that baseline oxidative conditions or cellular responsiveness may differ between these epithelial models. Oxidative stress is a central driver of aging (Liguori et al., 2018; Santos et al., 2024), contributing to DNA damage (Sánchez-Flores et al., 2017), mitochondrial impairment (Furman et al., 2025; Xu et al., 2025) and extracellular matrix degradation (Cai et al., 2023; Shin et al., 2023). The combination of glutathione (Aquilano et al., 2014), resveratrol (Yildiz et al., 2024), CoQ10 and PQQ provides complementary antioxidant mechanisms (Barcelos and Haas, 2019; Xie et al., 2025), both direct radical scavenging and indirect activation of endogenous defense pathways. From a longevity perspective, strengthening endogenous antioxidant capacity is more sustainable than relying solely on exogenous antioxidant buffering (Ristow and Schmeisser, 2014). Consistent with these observations, *GPX1* expression was significantly upregulated. In addition to its classical antioxidant role, *GPX1* is a key component of the glutathione-dependent defense system and plays a critical role in regulating ferroptosis, a form of iron-dependent cell death associated with oxidative damage. Recent studies have shown that modulation of the *GSH*-*GPX1* axis contributes to improved cellular survival and lifespan extension by limiting lipid peroxidation and ROS accumulation (Qian et al., 2025). Therefore, *GPX1* upregulation in our study may reflect activation of a broader cytoprotective and longevity-associated network. Enhanced *GPX1* expression suggests activation of intrinsic antioxidant defense systems, which aligns with longevity-promoting strategies aimed at improving stress resistance (Huang et al., 2025; Mandraffino et al., 2012; Qian et al., 2025). Interestingly, the broader transcriptional and redox response observed in hTERT-HME1 cells, compared to the more selective response in HaCaT cells, may reflect intrinsic differences in epithelial subtype-specific metabolism and redox sensitivity. Mammary epithelial cells are characterized by higher metabolic plasticity and NAD^+^ dependent signaling activity, which may render them more responsive to interventions targeting mitochondrial and redox pathways (Thomson et al., 2019). In contrast, keratinocytes such as HaCaT cells operate under a more tightly regulated differentiation-associated program with robust baseline barrier functions, potentially limiting the amplitude of inducible transcriptional responses (Dong et al., 2024). Therefore, the selective activation pattern observed in HaCaT cells may represent a more targeted adaptation rather than a weaker response.

Importantly, FOXO transcription factors are not merely stress-responsive regulators but are central drivers of longevity pathways (Martins et al., 2016; Zhao and Liu, 2021). They are also central regulators of oxidative stress resistance (Rodriguez-Colman et al., 2024), autophagy (Zhao et al., 2010) and metabolic adaptation (Jensen et al., 2011; Molaei et al., 2019), processes tightly linked to lifespan extension in multiple model organisms (Borch Jensen et al., 2017; Martins et al., 2016; Sánchez et al., 2023). In addition to evidence from *Caenorhabditis elegans*, studies in human cells have shown that FOXO transcription factors support oxidative stress resistance, antioxidant defense and autophagy, particularly in human chondrocytes. Thus, the observed upregulation of FOXO1A and FOXO3A may reflect activation of conserved stress-adaptive mechanisms relevant to human cellular homeostasis. (Li et al., 2024; Akasaki et al., 2014; Marinkovic et al., 2007; Rodriguez-Colman et al., 2024). In this context, the upregulation of *FOXO1A* and *FOXO3A* observed in our study suggests engagement of conserved pro-longevity signaling mechanisms rather than a transient adaptive response. *FOXO* activity is closely connected to NAD^+^ dependent signaling pathways, including sirtuin-mediated regulation (Keskin-Aktan et al., 2022; Yildiz et al., 2024). In the present study, this response was more pronounced in hTERT-HME1 cells, where *FOXO1A* and *FOXO3A* were significantly upregulated, in parallel with improvements in antioxidant status. In HaCaT cells, a significant increase in *FOXO3A* expression was also observed, although without corresponding changes in global TAS/TOS parameters, suggesting a more selective or context-dependent activation of stress-response pathways. Together, these transcriptional changes indicate that Elixea may influence conserved longevity-associated molecular circuits.

Notably, the concurrent upregulation of FOXO transcription factors and *GPX1* suggests a coordinated activation of evolutionarily conserved longevity pathways. While FOXO signaling enhances stress resistance and metabolic adaptation, *GPX1* contributes to redox stability and ferroptosis suppression. Together, these mechanisms may underlie the geroprotective potential of Elixea through integrated regulation of cellular survival and oxidative stress responses. This coordinated response is consistent with the concept that effective geroprotective interventions do not act through single pathways but rather through multi-layered regulation of stress resistance and redox homeostasis. These observations further support the notion that Elixea induces cell type-specific adaptive programs rather than a uniform transcriptional response.

In addition to redox and stress-response pathways, lipid homeostasis-related mechanisms also appear to be modulated by Elixea. Beyond its established role in systemic lipid transport, *APOE* has increasingly been recognized as a regulator of lipid homeostasis and barrier integrity in epithelial tissues (Rebeck, 2017; Santos et al., 2024). In these systems, lipids function not only as structural components but also as key determinants of membrane organization and permeability barrier maintenance, processes that are closely linked to mitochondrial lipid metabolism and fatty acid elongation pathways (Wikramanayake et al., 2022). In the present study, Elixea treatment resulted in a marked upregulation of *APOE* in both hTERT-HME1 (1.7-fold) and, more prominently, HaCaT cells (4.17-fold). This observation may reflect an adaptive cellular response aimed at maintaining membrane integrity and optimizing lipid redistribution under stress conditions. Considering that CoQ10 contributes to membrane stabilization against lipid peroxidation and that PQQ has been associated with improvements in barrier-related functions, the increase in *APOE* expression may be indicative of coordinated regulation of lipid handling and membrane remodeling processes (Barcelos and Haas, 2019; Mohamad Ishak et al., 2024).

Rather than representing a generic longevity-associated marker, *APOE* upregulation in this context may be linked to mechanisms that support epithelial structural integrity and tissue homeostasis, which are critical components of cellular resilience during aging (López-Otín et al., 2023).

The marked upregulation of *IL10* in hTERT-HME1 cells also warrants consideration in the context of inflammaging (Sbriscia et al., 2025). Chronic low-grade inflammation is a defining feature of aging and contributes to tissue dysfunction over time (Ferrucci and Fabbri, 2018; Franceschi and Campisi, 2014). Elevated *IL10* expression may indicate a shift toward an anti-inflammatory cellular phenotype, potentially mitigating inflammatory signaling that accelerates aging-related decline (Dagdeviren et al., 2017; Kinzenbaw et al., 2013). In contrast, *IL10* expression in HaCaT cells showed high variability and was not significantly altered, further supporting a cell type-specific response to Elixea. However, it is important to consider that excessive or sustained *IL10* signaling may also exert immunosuppressive effects, potentially leading to impaired immune surveillance or immune paralysis under certain conditions. Therefore, while the observed increase in *IL10* may be beneficial in the context of inflammaging suppression.

Notably, *TP53* and *RUVBL1* expressions remained largely unchanged in hTERT-HME1 cells, while *RUVBL1* was moderately upregulated in HaCaT cells. *RUVBL1* is a key component of chromatin remodeling complexes and has been implicated in DNA repair processes, particularly homologous recombination and maintenance of genomic stability (Clarke et al., 2017). Therefore, its upregulation in HaCaT cells may suggest activation of mechanisms supporting genome integrity under basal conditions. The absence of *TP53* induction in both cell types further indicates that Elixea does not provoke genotoxic stress or activate DNA damage responses under the tested conditions. This is particularly relevant in the context of longevity interventions, where enhancement of cellular resilience should occur without triggering damage-associated signaling pathways (Pandey et al., 2025).

Taken together, our results indicate that Elixea modulates key biological processes associated with aging, including redox regulation, stress response, and inflammatory balance, in a cell type-dependent manner. While hTERT-HME1 cells primarily exhibited improvements in antioxidant capacity and anti-inflammatory signaling, HaCaT cells showed enhanced viability and selective activation of stress-response pathways. Rather than acting as a generalized proliferative stimulus, Elixea appears to promote a molecular environment consistent with enhanced cellular resilience and maintenance of epithelial homeostasis.

However, this study has limitations. Experiments were conducted at a single concentration and time point, and functional measurements of mitochondrial respiration, intracellular NAD^+^ levels, or reactive oxygen species were not performed. Future studies should integrate metabolic flux analysis, long-term stress models, and *in vivo* validation to determine whether these transcriptional changes translate into durable improvements in tissue longevity. A further limitation of this study is that TAS/TOS measurements were performed only at the selected working concentration of 0.1 μg/mL, which was chosen based on the cell viability screening. Therefore, the present findings demonstrate redox modulation at a non-cytotoxic and biologically active concentration, but do not define a full dose-response relationship for redox homeostasis. Future studies should evaluate TAS/TOS responses across a broader concentration range to determine whether the redox effects of Elixea are concentration-dependent.

In summary, Elixea treatment at the selected working concentration modulated redox homeostasis and longevity-associated stress-response pathways in human epithelial cell models, particularly by enhancing antioxidant capacity and supporting an anti-inflammatory transcriptional profile in hTERT-HME1 cells. Given the short-term, *in vitro*, single-concentration design of this study, these findings should not be interpreted as direct evidence of geroprotective efficacy. Rather, they provide a preliminary basis for future studies evaluating whether Elixea or similar multi-component formulations may contribute to geroprotective effects under long-term, dose-dependent and physiologically relevant experimental conditions.

## Materials and methods

hTERT-HME1 and HaCaT cell lines were selected as complementary epithelial models for this study. hTERT-HME1 cells are non-tumorigenic, hTERT-immortalized human mammary epithelial cells that maintain near-normal gene expression profiles and have been widely employed in studies investigating redox homeostasis, antioxidant signaling, and aging-associated pathway activation in a non-cancerous epithelial context (Bodnar et al., 1998). HaCaT cells, a spontaneously immortalized human keratinocyte line, represent a well-established model for examining oxidative stress responses, epidermal barrier function, and longevity-associated signaling (Boukamp et al., 1988; Colombo et al., 2017). The use of two distinct epithelial cell types with different tissue origins and redox microenvironments allowed us to assess the cell type-dependent modulation of geroprotective pathways, thereby strengthening the translational relevance of our findings.

Furthermore, immortalized cell lines offer key practical advantages over primary cells in aging research, including stable and reproducible proliferative capacity across passages, absence of donor-to-donor variability, and the ability to maintain consistent experimental conditions over extended periods (Shitova et al., 2024). Although immortalized lines do not recapitulate replicative senescence, they retain the signaling competence of the pathways under investigation and are widely accepted models for mechanistic studies of aging-associated molecular networks and for evaluating the effects of bioactive compounds on cell viability, migration, and oxidative stress modulation (Karcioglu Batur et al., 2025; Lee et al., 2004; Shitova et al., 2024).

### Cell culture

Human hTERT-HME1 mammary epithelial cells (ATCC(CRL-4010), USA) and human HaCaT keratinocyte cells were used in this study. Cells were cultured in high-glucose DMEM (Sigma- Aldrich, ABD) supplemented with 10% fetal bovine serum (FBS; Sigma-Aldrich, ABD) and 1% penicillin–streptomycin (Gibco, USA). Cultures were maintained at 37 °C in a humidified incubator containing 5% CO2 (Wiggens, Germany).

Cells were grown in sterile 75 cm^2^ flasks and passaged at approximately 70% confluency. Culture medium was removed, cells were washed twice with sterile phosphate-buffered saline (PBS, pH: 7.4) and detached using 2 mL of 0.25% trypsin by incubating at 37 °C for 2–3 min. Cells were then resuspended in 8 mL of complete DMEM for further experimental procedures.

### Preparation of elixea solution

The Elixea supplement product (Kiperin, Türkiye) was dissolved in sterile PBS to obtain a stock concentration of 5 μg/mL. For all experiments, cells were treated with a final concentration of 0.1 μg/mL.

### Cell viability assay

Cell viability was evaluated using the 3-(4,5-dimethylthiazol-2-yl)-2,5-diphenyltetrazolium bromide (MTT) assay (EcoTech Biotechnology, Türkiye). hTERT-HME1 and HaCaT cells were seeded into sterile 96-well plates at a density of 1 × 10^4^ cells per well and allowed to attach 24 h under standard culture conditions. Following incubation, the culture medium was replaced with fresh medium containing increasing concentrations of Elixea (0.05–0.5 μg/mL) for hTERT- HME1 and HaCaT cells. Control wells received medium without treatment. After 24 h of treatment, 10 µL of MTT solution (5 mg/mL in PBS) was added to each well and cells were incubated for an additional 3–4 h at 37 °C in a humidified atmosphere containing 5% CO2. Subsequently, the supernatant was carefully removed, and the resulting formazan crystals were dissolved in 100 µL of dimethyl sulfoxide (DMSO). Absorbance was measured at 570 nm using a microplate reader (BioTek Instruments, ABD). Cell viability was expressed as a percentage relative to the control group, which was set to 100%. All experiments were performed in triplicate (n = 3).

### Wound healing assay

Cells were seeded into 6-well plates at a density of 4 × 10^5^ cells per well and incubated for 24 h under standard culture conditions to allow attachment and monolayer formation. Upon reaching approximately 90%–100% confluence, a linear scratch was generated across the cell monolayer using a sterile 100 µL pipette tip.

Detached cells were removed by gently washing the wells twice with PBS. Fresh complete medium containing 0.1 μg/mL Elixea was added to the treatment group, while control wells received fresh complete medium without treatment. Predefined regions along each scratch were marked to ensure imaging of the same field throughout the experiment. Images were captured at 0, 6, 20, 27, and 44 h for hTERT-HME1 and 0, 6, 25, 31 and 50 h for HaCaT using a fluorescent cell imager (ZOE™ Fluorescent Cell Imager, Bio-Rad Laboratories, Hercules, CA, United States).

Wound widths were quantified using ImageJ software (version 1.54 g). Measurements were performed using calibrated scale bars (100 µm), and the wound width was normalized to the 0 h time point (set as 100%) for each group and cells. Experiments were performed in triplicate (n = 3).

### Total antioxidant status (TAS) and total oxidant status (TOS) analyses

Cells were seeded in sterile Petri dishes and incubated for 24 h. After treatment with 0.1 μg/mL Elixea for 24 h, cells were washed twice with cold PBS on ice. Cells were lysed in 1 mL RIPA buffer (EcoTech Biotechnology, Türkiye) and scraped into microcentrifuge tubes. Lysates were centrifuged at 14,000 × g, and supernatants were collected for analysis.

TAS and TOS levels were measured using Rel Assay Diagnostics TAS and TOS kits (Mega Tıp, Türkiye) according to the manufacturer’s instructions. Absorbance values were measured using a spectrophotometer (BioTek Instruments, ABD) and converted to mmol/L (TAS) and µmol/L (TOS) based on kit calculation protocols. Experiments were performed in triplicate (n = 3).

### Total RNA isolation and cDNA synthesis

Cells were seeded into 6-well plates (4 × 10^5^ cells/well) and treated with 0.1 μg/mL Elixea for 24 h. Total RNA was isolated using TRIzol reagent (Invitrogen, ABD) according to the manufacturer’s protocol.

Briefly, 1 mL TRIzol was added per well after wash with PBS, and cells were lysed and transferred to nuclease-free tubes. Chloroform (250 µL) was added, samples were vigorously shaken for 15 s, incubated for 3 min at room temperature, and centrifuged at 12,000 × g for 15 min at 4 °C. The aqueous phase was transferred to a new tube, mixed with 500 µL isopropanol, and incubated for 10 min. RNA was pelleted by centrifugation at 12,000 × g for 10 min at 4 °C, washed with 75% ethanol, air-dried, and resuspended in 80 µL nuclease-free water.

RNA concentration and purity were determined using a NanoDrop ND-2000c spectrophotometer (Thermo Scientific, USA). RNA samples were normalized prior to cDNA synthesis. Purity was evaluated based on the A260/A280 absorbance ratio. One microgram of total RNA per sample was used for cDNA synthesis using the OneScript Plus cDNA Synthesis Kit (ABM, Canada) according to the manufacturer’s instructions.

### Gene expression analysis by qPCR

The effects of Elixea on the expression of *APOE*, *FOXO1A*, *FOXO3A*, *GPX1*, *IL10*, *RUVBL1* and *TP53* genes were evaluated by quantitative real-time PCR (qPCR). *GAPDH* was used as the housekeeping gene.

qPCR reactions were performed using BlasTaq 2X qPCR MasterMix (ABM, Canada) in a final volume of 20 µL containing 10 µL MasterMix, 0.5 µL forward primer, 0.5 µL reverse primer, 1 µL cDNA template, and nuclease-free water. A no-template control (NTC) was included in each run. Thermal cycling conditions were initial denaturation at 95 °C for 3 min, followed by 40 cycles of 95 °C for 15 s and 60 °C for 1 min. Amplification was performed using a CFX96 Real- Time System C1000 Touch Thermal Cycler (Bio-Rad, USA). Relative gene expression levels were calculated using the 2^−ΔΔCt^ method. All experiments were performed in triplicate (n = 3). Primer sequences are provided in Table 1.

**TABLE 1:** Forward and reverse primer sequences for *APOE*, *FOXO1A*, *FOXO3A*, *GPX1*, *IL10*, *RUVBL1* and *TP53* are presented. *GAPDH* was used as the housekeeping gene for normalization.

Gene	Primer pair sequences
***APOE***	F: 5′-GGGTCGCTTTTGGGATTACCTG-3′ R: 5′-CAACTCCTTCATGGTCTCGTCC-3′
***FOXO1A***	F: 5′-CTACGAGTGGATGGTCAAGAGC-3′ R: 5′-CCAGTTCCTTCATTCTGCACACG-3′
***FOXO3A***	F: 5′-TCTACGAGTGGATGGTGCGTTG-3′ R: 5′-CTCTTGCCAGTTCCCTCATTCTG-3′
***GAPDH***	F: 5′-CCACCCATGGCAAATTCC-3′ R: 5′-TGGGATTTCCATTGATGACAAG-3′
***GPX1***	F: 5′-GTGCTCGGCTTCCCGTCAAC-3′ R: 5′-CTCGAAGAGCATGAAGTTGGGC-3′
***IL10***	F: 5′-CCAGGGCACCCAGTCTGAGA-3′ R: 5′-AGCTTGGGGCATCACCTCCT-3′
***RUVBL1***	F: 5′-GAAGACAGAGGTGCTGATGGAG-3′ R: 5′-CTCTGTCTCACACGGAGTTAGC-3′
***TP53***	F: 5′-GTATTTCACCCTCAAGATCC-3′ R: 5′-CCAGGAGAAATCAAACAGAG-3′

### Statistics

All statistical analyses were performed using GraphPad Prism (version 11.0.0). Cell viability data were analyzed using one-way analysis of variance (ANOVA) followed by Tukey’s multiple comparison test. Wound healing, TAS/TOS, assay and gene expression data were evaluated using independent-samples Student’s t-test. Data are presented as mean ± standard deviation (SD). For cell viability assays, absorbance values were normalized to the control group, which was set to 100%. For wound healing analysis, measurements at each time point were normalized to baseline. (0 h), which was defined as 100%. Gene expression levels were calculated using the 2^−ΔΔCt^. method with GAPDH, as the reference gene and expressed as fold change relative to the control group. A p-value of less than 0.05 was considered statistically significant.

## Data Availability

The original contributions presented in the study are included in the article/supplementary material, further inquiries can be directed to the corresponding author.
